# Organelle genome composition and candidate gene identification for *Nsa* cytoplasmic male sterility in *Brassica napus*

**DOI:** 10.1186/s12864-019-6187-y

**Published:** 2019-11-06

**Authors:** Shi-Fei Sang, De-Sheng Mei, Jia Liu, Qamar U. Zaman, Hai-Yan Zhang, Meng-Yu Hao, Li Fu, Hui Wang, Hong-Tao Cheng, Qiong Hu

**Affiliations:** 1 Oil Crops Research Institute of Chinese Academy of Agricultural Sciences / Key Laboratory for Biology and Genetic Improvement of Oil Crops, Ministry of Agriculture and Rural Affairs, No.2 Xudong 2nd Road, Wuhan, 430062 People’s Republic of China; 2 National Key Laboratory of Crop Genetic Improvement, Huazhong Agricultural University, Wuhan, 430070 China

**Keywords:** Alloplasmic male sterility, Organelle genome sequencing, Somatic hybrid, Oilseed rape, *Sinapis arvensis*

## Abstract

**Background:**

*Nsa* cytoplasmic male sterility (CMS) is a novel alloplasmic male sterility system derived from somatic hybridization between *Brassica napus* and *Sinapis arvensis*. Identification of the CMS-associated gene is a prerequisite for a better understanding of the origin and molecular mechanism of this CMS. With the development of genome sequencing technology, organelle genomes of *Nsa* CMS line and its maintainer line were sequenced by pyro-sequencing technology, and comparative analysis of the organelle genomes was carried out to characterize the organelle genome composition of *Nsa* CMS as well as to identify the candidate *Nsa* CMS-associated genes.

**Results:**

*Nsa* CMS mitochondrial genome showed a higher collinearity with that of *S. arvensis* than *B. napus*, indicating that *Nsa* CMS mitochondrial genome was mainly derived from *S. arvensis*. However, mitochondrial genome recombination of parental lines was clearly detected. In contrast, the chloroplast genome of *Nsa* CMS was highly collinear with its *B. napus* parent, without any evidence of recombination of the two parental chloroplast genomes or integration from *S. arvensis*. There were 16 open reading frames (ORFs) specifically existed in *Nsa* CMS mitochondrial genome, which could not be identified in the maintainer line. Among them, three ORFs (*orf224*, *orf309*, *orf346*) possessing chimeric and transmembrane structure are most likely to be the candidate CMS genes. Sequences of all three candidate CMS genes in *Nsa* CMS line were found to be 100% identical with those from *S. arvensis* mitochondrial genome. Phylogenetic and homologous analysis showed that all the mitochondrial genes were highly conserved during evolution.

**Conclusions:**

*Nsa* CMS contains a recombined mitochondrial genome of its two parental species with the majority form *S. arvensis*. Three candidate *Nsa* CMS genes were identified and proven to be derived from *S. arvensis* other than recombination of its two parental species. Further functional study of the candidate genes will help to identify the gene responsible for the CMS and the underlying molecular mechanism.

## Background

Cytoplasmic male sterility (CMS) is a widely spread phenomenon in which the plant is unable to produce functional pollen [1, 2]. This phenomenon exists extensively in plant kingdom as a result of natural variations that follow the evolutionary path of mitochondrial genome rearrangement [3, 4]. CMS caused by natural variation of mitochondrial genome is usually called homoplasmic, which has been found in many crop species, such as WA CMS in rice [5], CMS-T in maize [6], *Don* CMS in radish [7], *Pol* CMS [8] and *Nap* CMS in rapeseed [9]. Some of the homoplasmic CMS has been successfully used as pollination control system for heterosis application, such as *Pol* CMS in rapeseed. However, drawbacks such as thermo-sensitive nature or lack of restoration in homoplasmic CMS systems have made the exploitation of alloplasmic CMS system desirable.

Interspecific or intergeneric hybridization has been proven efficient for the establishment of alloplasmic CMS in crops via either sexual or somatic hybridization, such as *Ogu* CMS derived from intergeneric hybridization of *Raphanus sativus* and *Brassica napus* [10], and *Tour* CMS from sexual hybridization of *Brassica juncea* and *Brassica tournefortii* [11]. Somatic hybridization is more advantageous compared to sexual hybridization for crossing genetically distant species by overcoming compatibility barrier as well as combining both organelle and nuclear genomes. Studies have revealed that alloplasmic CMS derived from cybrids retains a lot of donor mitochondrial genome sequences, as observed in Solanaceae [12] and Brassicaceae [13–15]. However, the composition of organelle genomes at whole genome level of a cybrid-derived alloplasmic CMS and the origin of its sterility gene have not been completely elucidated.

Previous studies have shown that plant CMS genes usually encode a protein with transmembrane domains and have a chimeric structure, containing coding sequences of known genes, e.g. those encoding ATPase, cytochrome-c oxidase, or ribosomal proteins etc. [16–18]. The most common method to identify the CMS-associated gene is to compare the differences of mitochondrial genes between the male sterile line and its maintainer lines at genomic, transcriptional and proteomic levels. For example, *orf288* as a specific novel chimeric ORF was identified to be *Hau* CMS candidate gene in *B. juncea* by comparison of genomic sequence and gene expression between the CMS line and its maintainer line using genome walking and Northern-blot analysis [19]. *OrfH79* in rice *HL* CMS [20] and *orf263* in rapeseed *Tour* CMS [11] were identified by using a similar approach. Other CMS genes, such as *orf138* of *Ogu* CMS in *B. napus* [21], *orf522* in sunflower CMS [22], *urf13* of CMS-T in maize [23] were detected by comparing the translated proteins in the CMS and fertile lines.

The advantage of next-generation sequencing (NGS) technology has allowed for convenient identification of CMS gene and a comprehensive investigation of all genes from both chloroplast and mitochondrial genomes. The size of plant chloroplast and mitochondrial genome is usually 120–160 kb and 200–2400 kb, respectively [24–26], both are much smaller than nuclear genomes. To date, there has been 3295 and 290 plant species with whole chloroplast or mitochondrial genomes sequenced, respectively (https://www.ncbi.nlm.nih.gov/genome/browse#!/organelles/). Compared to chloroplast genome, the mitochondrial genome is more unstable, with more long, short and tandem repeats, and thus is amenable to rearrangements. By using the whole genome sequencing approach, CMS-associated ORFs, including *orf463* in radish [7] and *orf507* in pepper [27] were identified.

Oilseed rape (*Brassica napus* L.) is the third largest oilseed crop worldwide, contributing about 27% of total edible plant oil production [28]. Significant heterosis has dramatically increased the yield of oilseed rape and will continue to contribute to the oilseed industry. CMS as the main pollination control system has been used in oilseed rape hybrid production for more than 20 years, including homoplasmic *Pol* CMS and alloplasmic *Ogu* CMS. With the development of embryo rescue and somatic hybridization technology, many alloplasmic CMS have been established [29–32]. Up to now, there are at least 30 alloplasmic male sterile systems in Brassicaceae crops, which greatly enrich the sterile cytoplasm type [33]. In *B. napus*, however, only *Pol* CMS and *Ogu* CMS are commonly used with some problems such as unstable sterility in *Pol* CMS and genetic drag of Radish segment in *Ogu* CMS restorers. *Nsa* CMS was identified to be a novel *B. napus* alloplasmic male sterility system derived from somatic hybridization of *B. napus* cv. Zhongshuang 4 and *Sinapis arvensis* var. Yeyou 18 [34]. The *Nsa* CMS line is essentially different from other rapeseed CMS systems such as *Ogu*, *Nap*, *Pol*, *Tour* and *Hau*, based on their origins, genetic, morphological, cytological and molecular characterization [35–37]. Compared with the most commonly used *Pol* CMS, *Nsa* CMS is less susceptible to temperature with stable sterility [29]. Progress has been made on hybrid variety development with *Nsa* CMS and several new hybrids are on the way for registration. In order to characterize the organelle genome composition and identify candidate CMS-associated genes of *Nsa* CMS, we sequenced the complete organelle genomes of *Nsa* CMS line and its iso-nuclear maintainer line of *B. napus* cv. Zhongshuang 4 which is the original parental line used in somatic hybridization by employing Roche/454 pyro-sequencing technology. Comparative analysis of the organelle genome sequences of the two lines together with *Sinapis arvensis* var. Yeyou 18, which is the other parental species for *Nsa* CMS, resulted in the identification of candidate *Nsa* CMS-associated gene and the composition of organelle genomes in *Nsa* CMS. Our results will lay a foundation for elucidating the mechanisms of plant CMS and give insights into the formation of alloplasmic male sterility system.

## Results

### Assembling of chloroplast genomes

The Roche 454 sequencing platform generated 46.3 M bp and 33.4 M bp of sequence reads for *Nsa* CMS and Zhongshuang 4, respectively. In addition, 546,642 and 618,772 clean reads were generated for Zhongshuang 4 and *Nsa* CMS by Miseq sequencing. The reads were firstly assembled into 5 contigs in each line with the coverage of 38.8× and 52.3×, respectively. PCR amplification of contig ends showed that there was no missing base between the contigs, and the break between contigs was caused by inverted repeat (IR) sequence. The chloroplast genomes of *Nsa* CMS and maintainer lines were individually assembled into a single, circular mapping molecule with a size of 153,449 bp (Genbank no. MN428073), and 153,458 bp (Genbank no. MN428074), and G + C content of 36.37 and 36.35%, respectively.

**Fig. 1:**
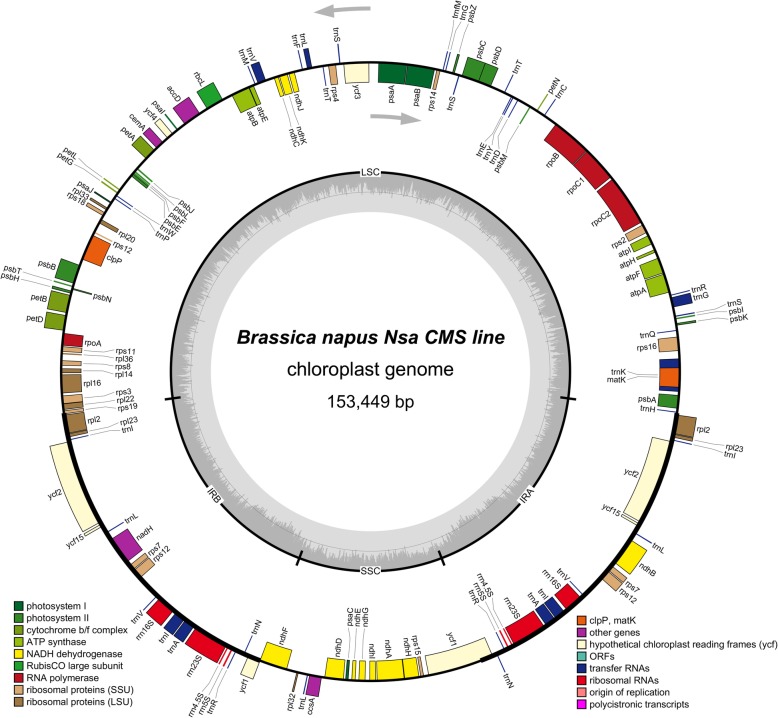
Map of *Nsa* CMS chloroplast genome. Thick lines represent the inverted repeats (IRa and IRb), which separate the genome into large and small single copy region (LSC and SSC)

The chloroplast genomes of both lines have similar structure, containing a pair of inverted repeat (IR) regions, which were divided by short single copy (SSC) and long single copy (LSC) regions (Fig. 1). The chloroplast genome possesses a total of 129 genes, including 4 rRNA genes, 30 tRNA genes, and 79 protein-coding genes (Table 1). Within the IR regions, there are 16 duplicate genes, and *rps19* gene spans the IR and SSC regions.

**Table 1:** Characteristics of chloroplast genomes of *Nsa* CMS, Zhongshuang 4 and *S. arvensis*

Feature	Nsa CMS	Zhongshuang 4	*Sinapis arvensis*
Genome size (bp)	153,449	153,458	153,590
GC content(%)	36.37%	36.35%	36.31%
Protein coding genes	79	79	79
ycfs	5	5	5
tRNA genes	30	30	29
rRNA	4	4	4

### Assembling of mitochondrial genomes

The reads data generated from mitochondrial DNA of *Nsa* CMS and Zhongshuang 4 were assembled into 16 and 9 contigs with the coverage depth of 191× and 195×, respectively. The average contig length of *Nsa* CMS and Zhongshuang 4 was 15.56 kb and 24.53 kb, respectively. The gap length of *Nsa* CMS genome between contigs was 0–500 bp, and that of Zhongshuang 4 genome was all less than 50 bp.

**Fig. 2:**
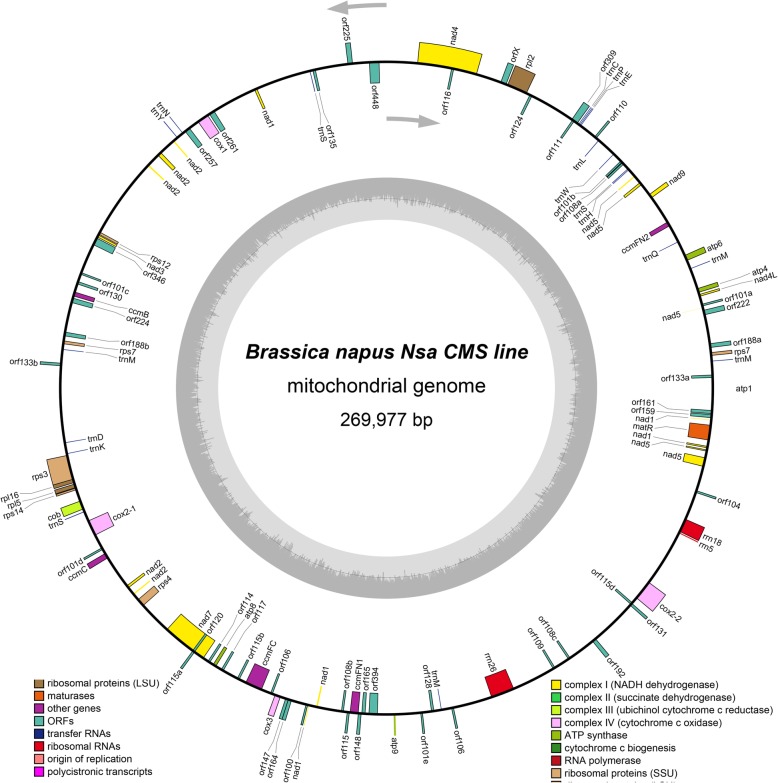
Map of *Nsa* CMS mitochondrial genome. The features of the transcriptionally clockwise and counter-clockwise strands are indicated on the inside and outside of the circle, respectively

After filling in the gaps, the mitochondrial genomes of *Nsa* CMS and its maintainer were each assembled into a single, circular mapping molecule with a size of 269,977 bp (Genbank no. MN443182) and 221,862 bp (Genbank no. MN428072), and G + C content of 45.08 and 45.18%, respectively (Fig. 2). The two mitochondrial genomes shared similarities on gene composition, both consisting of 34 protein coding genes, 18 tRNA and 3 rRNA genes (Table 2).

**Table 2:** Characteristics of mitochondrial genomes of *Nsa* CMS, Zhongshuang 4 and *Sinapis arvensis*

Feature	Nsa CMS	Zhongshuang 4	*Sinapis arvensis*
Genome size (bp)	269,977	221,862	240,024
GC content (%)	45.08%	45.18%	45.23%
Protein coding gene	34	34	33
ORFs	49	45	45
tRNA gene	18	18	18
rRNA gene	3	3	3

Sequence alignment showed that the mitochondrial genome of Zhongshuang 4 was 9 bp larger than previously reported *B. napus* cv. “Westar” (Genbank accession number: AP006444.1), and only 31 single nucleotide polymorphisms (SNPs) were detected between the two mitochondrial genomes. One 2427 bp forward repeat sequence was found in mitochondrial genome of Zhongshuang 4, which means a tripartite mitochondrial genomic structure existed in this mitotype [38]. Based on the repeat sequence in the mitotypes, the mitochondrial is inferred as containing one master circle accompanied by two smaller circles of 124,910 bp and 69,952 bp in size, which was similar with Westar [39].

### Comparison of protein-coding gene in the mitochondrial genome

Functional genes are conserved in the mitochondrial genome and their evolution is very slow among different plant species [40, 41]. The differences of protein sequences are important candidates for functional analysis of CMS-associated genes as well as revealing the molecular mechanisms of CMS. Based on our sequence data, most protein-coding genes in the mitochondrial genome of *B. napus* and *S. arvensis* are identical, especially in size, except *rps3* and *cox2–2* (S, B, Nsa is prefixed to the names of genes/ORFs in *S. arvensis*, *B. napus* and *Nsa* CMS, respectively). For instance, in the 2nd exon of *Brps3*, there is a 33 bp insertion in *Srps3* and *Nsarps3*, but not in *Nrps3*. Another case is *cox2–2*, which is absent in *S. arvensis* but present in both *B. napus* and *Nsa* CMS.

In addition, there were 26 single nucleotide polymorphism (SNPs) scattered among 12 protein-coding genes. Among these SNPs, 10 were synonymous (*nad3*, *rps12*, *rps7*, *orfX*, *cox1*, *atp1*) and 16 were non-synonymous (Table 3). Most of these SNPs were transitions.

**Table 3:** Protein coding gene difference between mt genome of Zhongshuang 4 and *Nsa* CMS

*Gene*	Zhongshuang 4-Nsa CMS			
	nucleic acid^a^	amino acid^b^	Mutation type	SNP type
*rpl5*	515C-T	172C-R	N	transition
*atp1*	405C-A	N	S	transversion
	1153G-A	385A-T	N	transition
*ccmFN1*	397C-A	133S-R	N	transversion
	997C-T	333 L-F	N	transition
*cox1*	109C-T	N	S	transition
	111 T-G	N	S	transversion
	129A-G	N	S	transition
	786 T-G	N	S	transversion
*nad4*	836C-T	279S-F	N	transition
*orfX (tatC)*	15C-T	N	S	transition
	73C-T	25R-W	N	transition
	220A-G	74I-V	N	transition
	355C-T	119R-C	N	transversion
	550 T-C	184C-R	N	transition
	641C-T	214P-F	N	transition
*rpl2*	98G-A	33R-Q	N	transition
	128G-A	43R-K	N	transition
	845G-C	282R-T	N	transversion
*ccmFN2*	380 T-A	127 L-S	N	transversion
*rps7*	276C-T	N	S	transition
*matR*	1273G-A	425G-S	N	transition
	1436G-A	479R-Q	N	transition
*rps12*	334A-C	N	S	transversion
	336A-C	N	S	transversion
*nad3*	265 T-C	N	S	transition

^a^Location of base mutation

^b^Location of amino acid mutation

S, synonymous; N, Non-synonymous

### Composition of *Nsa* CMS organelle genome

**Fig. 3:**
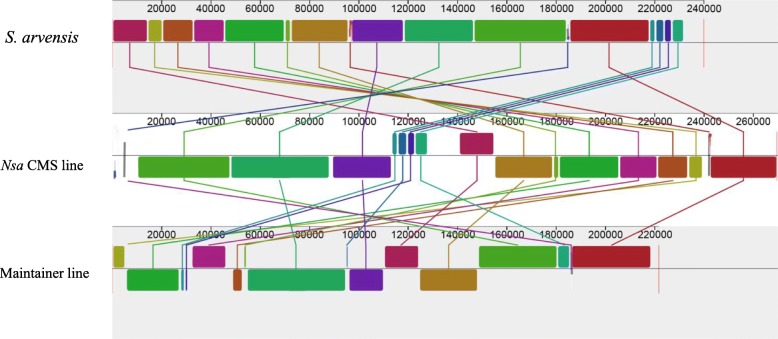
Comparative diagrams of *Nsa* CMS, Zhongshuang 4 and *Sinapis arvensis* mitochondrial genomes. Different blocks are assigned with different color in each mitochondrial genome, and the corresponding line that connects two blocks indicates high homology of these two blocks. Direct or reverse transcript orientation is indicated above and below the central line, respectively

To elucidate the formation and organelle composition of *Nsa* CMS system that established by somatic hybridization, we performed a comparative analysis of the whole organelle genome of *Nsa* CMS and its parental lines, Zhongshuang 4 (maintainer line) and *S. arvensis*. The organelle genome of *S. arvensis* was previously sequenced (Genbank no. KM851044). Although *Nsa* CMS mitochondrial genome showed some collinearity with the maintainer line, the collinear segments were short and scattered in the genome (Fig. 3). A much higher collinearity of *Nsa* CMS mitochondrial genome with that of *S. arvensis* was inferred. Sequence alignment using BLASTN showed that the sequence coverage of Zhongshuang 4 mitochondrial genome to *Nsa* CMS mitochondrial genome was 86%, with an identity of 99.85%. In contrast, the sequence coverage of *S. arvensis* mitochondrial genome to that of *Nsa* CMS was 93%, with an identity of 99.93%.

**Fig. 4:**
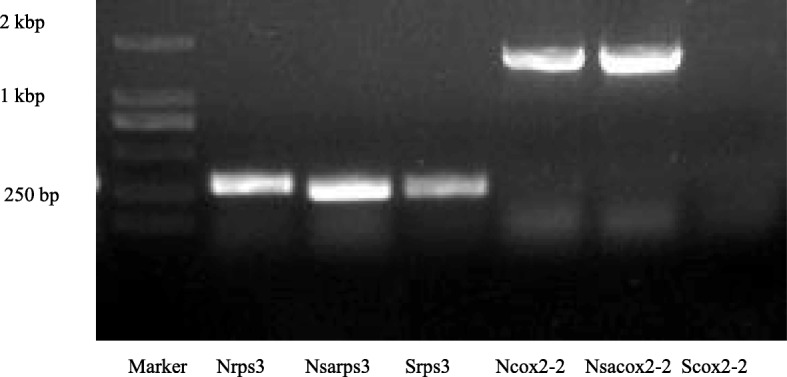
PCR amplification of *rps3* and *cox2–2* gene from Zhongshuang 4, *Nsa* CMS and *S. arvensis*. B-, Nsa-, S- represent PCR product of the corresponding gene from from Zhongshuang 4, *Nsa* CMS and *S. arvensis*, respectively

We also compared coding sequences of the mitochondrial genome of the three materials (Table 4). Except for 16 genes which were completely consistent among these three materials, there were 18 coding genes that were different. Among the different genes, 13 were from *S. arvensis* and 5 genes were from *B. napus*. In addition, the sequence of *rps3* and *cox2–2* are different in the mitochondrial genomes of *B. napus* and *S. arvensis*, and the deletion in *rps3* and *cox2–2* were present in *Nsa* CMS, which means that *Nsa* CMS has *rps3* gene from *S. arvensis* cv. “Yeyou 18” and *cox2–2* gene from Zhongshuang 4. To verify these genomic differences, we designed two primer pairs specific for *rps3* and *cox2–2*, respectively. PCR amplification of *rps3* gene showed that an identical fragment was generated from *Nsa* CMS and *S. arvensis*, but a shorter fragment from Zhongshuang 4. Meanwhile, an identical fragment of *cox2–2* gene was amplified from *Nsa* CMS and Zhongshuang 4, but no product was obtained from *S. arvensis* (Fig. 4). Sequencing of the amplified fragments confirmed the deletion in *rps3* gene from *Nsa* CMS and *S. arvensis*, and the existence of *cox2–2* in *Nsa* CMS and Zhongshuang 4.

**Table 4:** Protein coding gene difference among *Nsa* CMS, Zhongshuang 4 and *S. arvensis* mt genomes

Gene	*S. arvensis-Nsa CMS*		*B. napus-Nsa CMS*	
	nucleic acid^a^	amino acid^b^	nucleic acid^a^	amino acid^b^
*rpl5*	–	–	515C-T	172C-R
*atp1*	–	–	405C-A	N
	–	–	1153G-A	385A-T
*ccmFN1*	–	–	397C-A	133S-R
	–	–	997C-T	333 L-F
*nad7*	1079 T-C	360F-S	–	–
*rps4*	711 T-C	S	–	–
*nad2*	367 T-C	123C-R	–	–
*cox1*	–	–	109C-T	S
	–	–	111 T-G	S
	–	–	129A-G	S
	–	–	786 T-G	S
*nad4*	–	–	836C-T	279S-F
*orfX (tatC)*	–	–	15C-T	S
	–	–	73C-T	25R-W
	–	–	220A-G	74I-V
	–	–	355C-T	119R-C
	–	–	550 T-C	184C-R
	–	–	641C-T	214P-F
*rpl2*	–	–	98G-A	33R-Q
	–	–	128G-A	43R-K
	–	–	845G-C	282R-T
*nad5*	242C-T	81P-L	–	–
*ccmFN2*	–	–	380 T-A	127 L-S
*rps7*	–	–	276C-T	S
*matR*	–	–	1273G-A	425G-S
	–	–	1436G-A	479R-Q
*rps12*	–	–	334A-C	S
	–	–	336A-C	S
*nad3*	–	–	265 T-C	S
*cox2–2*	379C-T	127R-W	–	–
*rps3*	–	–	33 bp	11AA

^a^Location of base mutation. ^b^Location of amino acid mutation. S, synonymous. -, *Nsa* CMS has the same DNA or amino acid sequence as *B.napus* or *S. arvensis*

Four sequences with 9432 bp, 7383 bp, 2427 bp and 1592 bp length were present in *Nsa* CMS mitochondrial genome as repeat sequences, with the first three were reverse repeats and the last one is forward repeats. However, repeats with 7383 bp and 2427 bp length appeared as forward repeats in both *S. arvensis* and Zhongshuang 4 mitochondrial genomes. The 9432 bp and 7383 bp length repeats contained two short repeat sequences, indicating that the inverted repeats can be easily converted into another direction. More repeat sequences may be the reason for the greater number of gaps in the mitochondrial genome of *Nsa* CMS than those of *B. napus* and *S. arvensis*.

Comparative analysis of chloroplast genome showed that *Nsa* CMS had a higher collinearity with Zhongshuang 4 than *S. arvensis*. Sequence alignment showed that the identity between chloroplast genomes of *Nsa* CMS and Zhongshuang 4 was 99.96%, and those of *Nsa* CMS and *S. arvensis* was 97.6%, possessing very high collinearity (Additional file 1). Apart from 60 SNPs and 50 gaps (0–5 bp delations or insertions), the chloroplast genome of *Nsa* CMS was very similar to that of the maintainer line without any structural difference. In contrast, 521 SNPs and 632 gaps between the chloroplast genomes of *Nsa* CMS and *S. arvensis* were identified. These results indicate that the chloroplast genome of *Nsa* CMS was derived directly from its *B. napus* parent Zhongshuang 4.

### Identification of CMS-associated ORFs

**Fig. 5:**
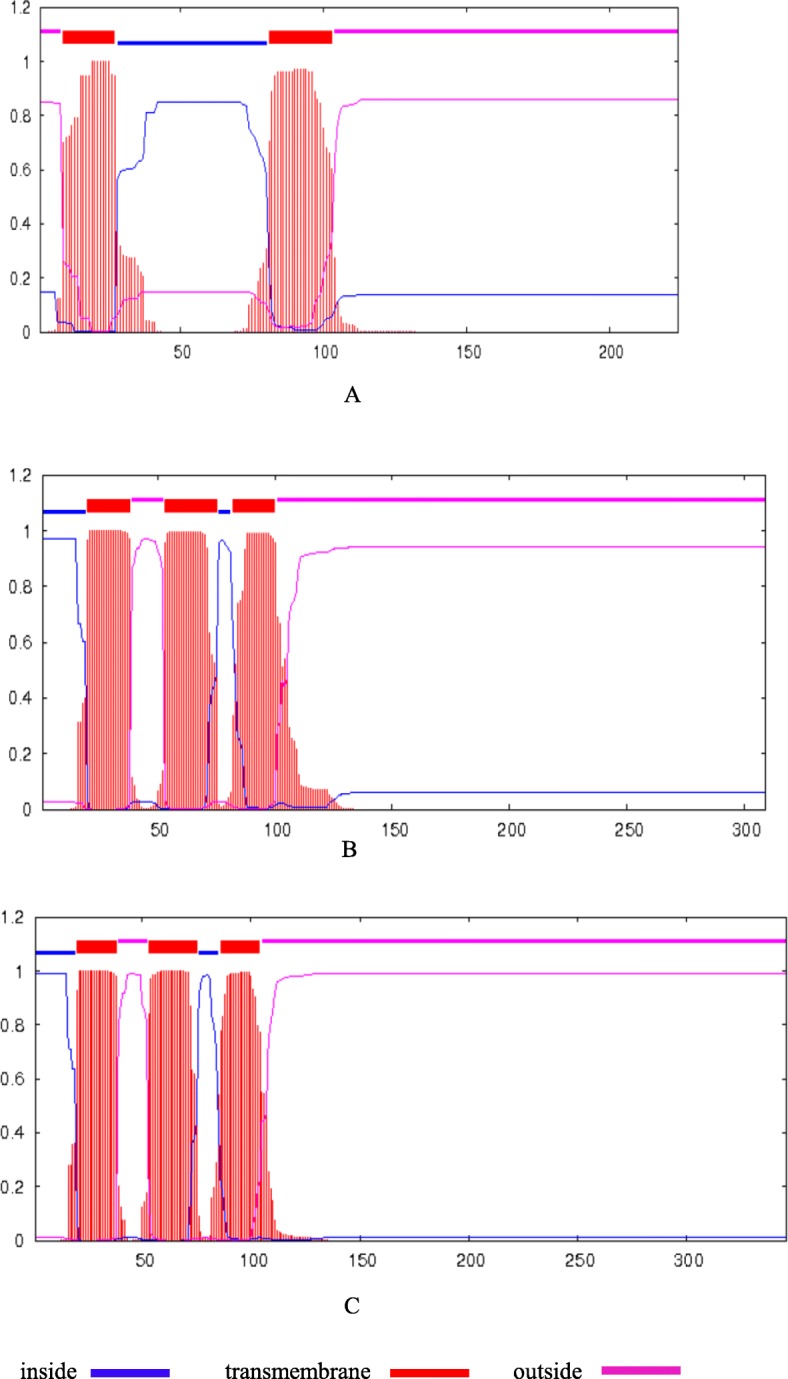
Transmembrane domain prediction of three candidate CMS gene coding proteins. The output of the TMHMM server shows the location and probability associated with the predicted transmembrane domain. **a**: ORF224, **b**: ORF309, **c**: ORF346

Forty-nine ORFs encoding proteins with over 100 amino acids have been found in *Nsa* CMS mitochondrial genome. Among them, 16 ORFs were specifically detected in *Nsa* CMS but not in the maintainer line. Transmembrane prediction revealed that 11 of the *Nsa* CMS specific ORFs possessed transmembrane domains (Table 5, Fig. 5). Three of the *orfs*, *orf224*, *orf309* and *orf346* contained a chimeric structure. The *orf224* is a 675 bp gene containing a 175 bp homologous fragment with 98% identity to *atp8*. Both *orf309* and *orf346* are chimeric with *cox1*, in which a 133 bp sequence was found with 98% identity.

**Table 5:** Specific ORFs in *Nsa* CMS mitochondrial genome

Gene name	Gene length	Transmembrane	Chimeric gene
*orf108a*	327	1	–
*orf133a*	402	0	
*orf110*	333	1	–
*orf309*	930	3	*cox1*
*orf257*	774	6	–
*orf346*	1041	3	*cox1*
*orf101b*	306	0	–
*orf130*	393	1	–
*orf224*	224	2	*atp8*
*orf133b*	402	0	–
*orf106*	321	1	–
*orf115a*	348	2	–
*orf170*	513	1	–
*orf394*	1185	0	–
*orf192*	579	1	–
*orf131*	396	0	–

Note: The number of transmembrane columns represents the number of transmembrane structures

**Fig. 6:**
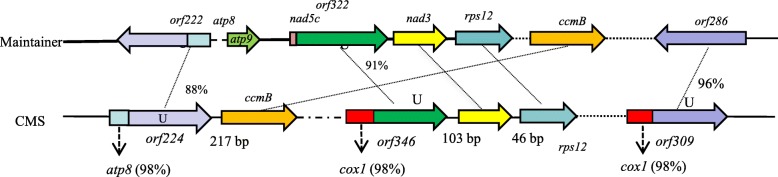
The organization of mitochondrial genome regions associated with candidate sterility gene for *Nsa* CMS and Zhongshuang 4 mitotypes. The percentage represents identity

Almost all identified plant CMS-associated ORFs were chimeric genes, mostly located upstream or downstream functional genes, encoding components of the electron transport respiratory chain, and possessed transmembrane domains [18]. According to the mitochondrial genome sequencing data, *orf224*, *orf346* and *orf309* are all chimeric genes, of which *orf224* and *orf346* located upstream known genes in *Nsa* CMS (Fig. 6). *Orf346* is located upstream *nad3* and *rps12*, with only 103 bp distance from *nad3*. *ccmB* is located 217 bp downstream *orf224*, but there is no gene located near *orf309*. All the three *orfs* existed in *S. arvensis* but not in the maintainer.

*Orf224* in *Nsa* CMS (*Nsaorf224*) has 93% sequence identity to that of *Pol* CMS associated gene *orf224* (*Polorf224*) and there are 27 different amino acids between them. A corresponding *orf222* in the maintainer line which is also chimeric with *atp8*, was found to have 88% similarity with *Nsaorf224*, and 84% similarity with *Polorf224*. *Orf286* in the maintainer line is homologous to *orf309*, with 96% sequence similarity. *Orf322* in the maintainer line is homologous to *orf346* with 91% sequence similarity.

**Fig. 7:**
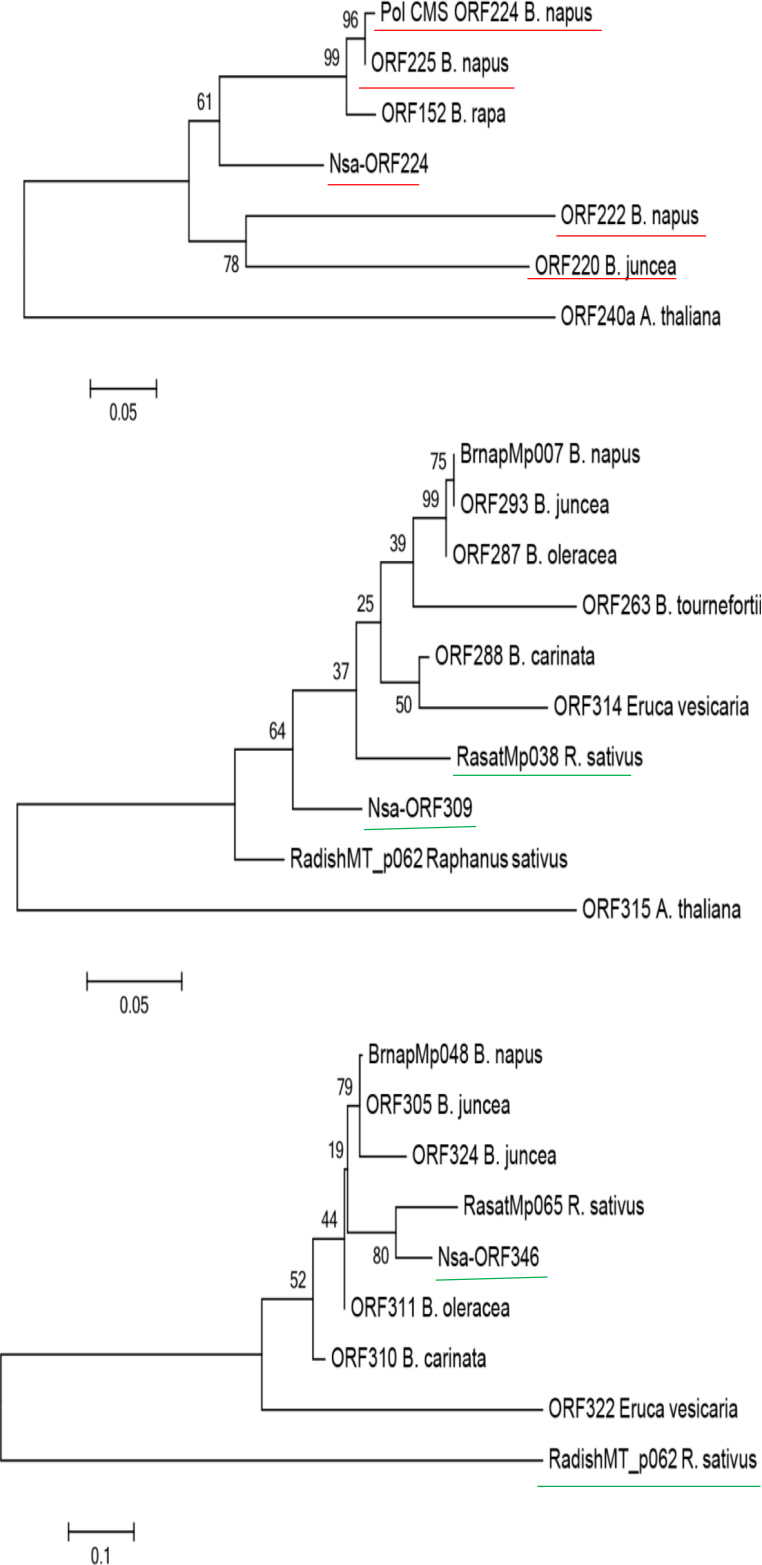
Phylogenetic analysis of three CMS candidate genes with their homologous genes. The red line and green line represent the corresponding gene chimeric with *atp8* and *cox1*, respectively

Blast search for homologous of the three *orf*s from Genbank resulted in 6, 9 and 8 homologous for *Nsaorf224*, *Nsaorf309* and *Nsaorf346*, respectively. Phylogenetic analysis showed that the relatedness between these three *orf*s and the homologous from *B. napus* was quite distant. *Nsaorf224* was not grouped with any of the homologous in *Brassica* species. *Orf309* and *orf346* showed a closer genetic relationship with homologous in *R. sativus* than those in *Brassica* species, and homologous sequence analysis shows that the chimeric structure of *cox1* in *orf309* and *orf346* only existed in *R. sativus* among Brassicacea species (Fig. 7).

## Discussion

### Origin of *Nsa* CMS organelle genome

There has been a large number of study on cytoplasmic source of alloplasmic CMS in plants [12, 42–44]. Plenty of work has been done on the analysis of heterologous cytoplasm, but only a few studies reported on the origin of the cytoplasmic genome of heterologous CMS line. For example, the mitochondrial of the first alloplasmic male sterility system in tobacco were confirmed to be a fusion type, whereas the chloroplasts came from a single parent [45, 46]. Alloplasmic male sterility system derived from somatic hybridization between *B. napus* and *A. thaliana* contained a mixed mitochondrial composition from both parents [42]. The SW18 mitochondrial genome has the whole circular genome of *B. napus* cv. “Westar” with about 60% redundancy, and four partial fragments of Kosena radish [30]. Mitochondrial genome sequencing showed that the exogenous sequence was mostly derived from the donor parent (radish) of somatic hybrids in *Ogu* CMS [44]. Alloplasmic CMS line of durum wheat contained several heterologous regions [47]. From the perspective of current research, plants regenerated from fused cells and their progeny usually inherited the chloroplasts from only one of the parents, while the mitochondrial genome is recombined from both parents [12, 30, 44, 48].

As a novel alloplasmic CMS system developed by somatic hybridization between *B. napus* and *S. arvensis* [34], *Nsa* cytoplasm was proven to be different from other CMS systems in *B. napus* and contains mitochondrial genomic fragments from both parental species by RFLP markers [36]. In this study, by comparative analysis of the whole organelle genome, we found that *Nsa* CMS chloroplast genome was completely derived from the *B. napus* parent, and its mitochondrial genome was derived from the recombination of *B. napus* and *S. arvensis*. However, the higher collinearity of *Nsa* CMS mitochondrial genome with *S. arvensis* revealed that the majority of *Nsa* CMS mitochondrial genome was derived from *S. arvensis*. This is consistent with previous conclusion that cybrid progeny inherit chloroplasts from only one of the parents, while the mitochondrial genome is recombined from both parents [12, 30]. A large number of repeat sequences in mitochondria are the source of mitochondrial recombination, whereas there are much fewer repeat sequences in the chloroplast genome. This might be the reason that the chloroplast can inherit steadily and the mitochondrial genomes undergo recombination in the process of somatic cell fusion.

### NGS is an efficient approach to acquire CMS-associated gene

Comparative analysis of the CMS line with its maintainer line on mitochondrial gene structure, expression and translation products has become the conventional approach to identify CMS gene. For example, using genomic walking and Northern-blot analysis, the *Hau* CMS *orf288* in *B. juncea* [19], *orfH79* in rice *HL* CMS [20] and *orf263* in rapeseed *Tour* CMS [11] were identified. Analysis of protein difference has also led to the identification of *orf138* from *B. napus Ogu* CMS [21], *orf522* from sunflower CMS [22], *urf13* of CMS-T from maize [23] and *orf147* from *Cajanus cajan* A4 CMS [49].

In recent years, with the development of genome sequencing technology, many CMS genes were identified by directly sequencing and comparison the whole mitochondrial genome of CMS and its maintainer lines, such as *orf182* from rice D1 CMS [50], *orf507* from peppers [27], *orf113* from rice RT98A CMS [51], and *orf463* in radish [7]. This approach is much easier and straighter forward than the conventional ones due to the small size of plant mitochondrial genome, which varied from 200 to 2400 kb [44, 52, 53]. In this study, we identified three ORFs specific to the CMS line with transmembrane domain and chimeric structure. All of them contained the typical structure of plant CMS genes and are very likely to be the candidate CMS genes for *Nsa* CMS.

### The origin of *Nsa* CMS gene

As there is a large number of repetitive sequences in the mitochondrial genome, rearrangement or recombination events of the genome usually occur during species evolution [47]. The instability of mitochondrial genome has been reported the reason for generation of new ORFs, especially those related to CMS [3, 26]. Although, somatic hybridization could accelerate the process of recombination in mitochondrial genome and *Nsa* CMS mitochondrial genome derived from recombination events between *S. arvensis* and *B. napus*, three candidate sterile genes identified were not formed by recombination of the two parental species in this study. All the three candidate genes were directly derived from *S. arvensis* mitochondrial genome without any sequence variation.

Previous studies showed that genes related to crop CMS were mainly derived from alien or ancestral species, such as *orf138* in *B. napus* from *R. sativus*, *orfH79* and *WA352* in *O. sativa* from wild rice [54, 55],, *orf263* in *B. juncea* from *B. tournefortii* [11]. Even in CMS systems derived from cybrids, the source of CMS genes was proven to be directly from the donor parental species, such as Brassica CMS genes *orf138* and *orf125,* which originated from radish, *orf263* in *B. juncea* from *B. tournefortii* [11, 30, 44].

To date, almost all identified CMS-associated ORFs possessed chimeric genes and transmembrane domains [18]. Three *Nsa* CMS-associated ORFs also possess chimeric structure. However, there are few homologous genes with *Nsa* CMS-associated genes. It signifies that the mitochondrial genes have considerable stability during evolution and alloplasmic male sterile is caused by incompatibility between cytoplasm and nucleus. The candidate CMS genes identified in our study should also be a result of mitochondrial genome rearrangement, which occurred in *S. arvensis* or its ancestral species, other than recombination of parental genomes in cybrids.

### Structure of CMS gene and its functional prediction

We conducted extensive sequence comparison between *B. napus* and *Nsa* CMS mitochondrial genomes to search for functional alterations of genes that were responsible for the CMS phenotype. It is noted that several partial subunits of the respiratory chain complex and cytochrome c assembly protein, including ATP1, NAD4, CCMFN1, CCMFN2 encoded by mitochondrial genome were different between *Nsa* CMS and its maintainer line. Any of these altered proteins may interfere with the electron transfer chain (mtETC), weakening energy supplies and stalling pollen development [56]. We also observed amino acid variations among RPL5, ORFX (TATC), RPL2, MATR, RPS3 coded by *Nsa* CMS mitochondrial genome. Almost all the mitochondrial functional genes play an absolutely necessary role in maintaining the completeness of mitochondria and metabolism, and there have not been reports of CMS caused by mutation of functional mitochondrial genes so far. Thus, we think that these differences on functional genes may not directly associate with cytoplasmic male sterility. Whether these variant genes are related to the sterility regulation in *Nsa* CMS requires further study.

To date, almost all identified CMS genes are novel chimeric open reading frames (ORFs). Studies have shown that at least 10 mitochondrial genes, involving electron transfer chain (mtETC) pathways, such as *cox1*, *atp8*, and *atp6* were frequently involved in the formation of CMS genes [18]. In rapeseed, *orf222* for CMS-Nap and *orf224* for CMS-Pol encode proteins with an N terminus similar to ATP8 and the remaining sequence of unknown origin [8, 9]. In Pepper CMS-Peterson and Sugar beet CMS-Owen, the chimeric *orf456* and *preSatp6* encode a protein with a segment of ATP6 at the N terminus [57, 58]. Rice *orf79* for CMS-BT and *orfH79* for CMS-HL [59, 60], and radish *orf463* for CMS-Don [7] encode small proteins with an N terminus similar to COX1 and the remaining sequence of unknown origin. These CMS genes consisting of portions of some essential mitochondrial genes may competitively interact with the mtETC complexes, and result in decreased ATP production [18].

Also, all the proteins encoded by plant CMS genes possess transmembrane domains, such as URF13 for maize CMS-T [61], ORF138 for *B. napus* CMS-Ogu [21], ORF79 for rice CMS-BT [60], ORFH79 for rice CMS-HL [59], and ORF224 for rapeseed CMS-Pol [8]. The CMS proteins may integrate into the inner mitochondrial membrane, destroying the proton gradient and affecting ATP synthesis. For example, ORFH79 in *HL* CMS, interacts with subunit P61 of mtETC complex III, impairing the activity of complex III and resulting in decreased ATP production [59]. This explains the necessity of the sterility proteins being membrane proteins, otherwise they are unable to interfere with energy synthesis.

In the present study, three ORFs with transmembrane and chimeric structures were identified in the mitochondrial genome of *Nsa* CMS. Of which *orf224* is chimeric with *atp8*, and both *orf309* and *orf346* are chimeric with *cox1*. The chimeric segments may interact with corresponding functional genes involved in mtETC leading to fertility abortion of pollen. Therefore, these three ORFs are excellent candidate sterility genes of *Nsa* CMS. Further functional studies of these candidate genes are required to identify *Nsa* CMS associated gene and understand the sterility regulation mechanism in *Nsa* CMS.

## Conclusions

The complete organelle genome sequences of *Nsa* CMS and its maintainer lines in *B. napus* were obtained and characterized. Comparative analysis of whole organelle genomes of *Nsa* CMS line and its parents showed that *Nsa* CMS mitochondrial genome was derived from the asymmetrical fusion of its parent lines, but *Nsa* CMS chloroplast genome was derived from the *B. napus* parent. Three ORFs, *orf224*, *orf309* and *orf346*, coding for hypothetical proteins with a chimeric structure and transmembrane domain were identified as the candidate genes for *Nsa* CMS. Their existence in both *Nsa* CMS and *S. arvensis* revealed that the candidate CMS genes were not caused by rearrangement of mitochondrial genome during somatic hybridization. All of the candidate sterile ORFs were derived from *S. arvensis* and formed either in *S. arvensis* or its progenitor.

## Materials and methods

### Plant materials

*Nsa* CMS line was produced by repeatedly backcrossing cybrids from somatic hybridization of *B. napus* cv. “Zhongshuang 4” and *S. arvensis* cv. “Yeyou 18” with Zhongshuang 4. Seeds of Nsa CMS, Zhongshuang 4 and *S. arvensis* were harvested from the experimental field of Oil Crops Research Institute, Chinese Academy of Agricultural Sciences in Wuchang, Hubei.

### Isolation of organelle DNA

Total DNA was isolated from the organelles by employing discontinuous sucrose gradient as described by Chen et al. (2011) [38]. Seven days old etiolated seedlings grown at 25 °C were used to extract Organelle DNA. CTAB method was employed for total DNA extraction.

### Cytoplasmic genome composition identification

The primers were designed according to the sequence differences of *rps3* and *cox2–2* genes in *Nsa* CMS and maintainer lines. The primer sequences were listed in Additional file 2. PCR program was as following: 94 °C 30 s, 56 °C 30 s, 72 °C 1 min, 32 circles.

### Genome sequencing and sequence analysis

DNA samples with good quality were used to construct sequencing libraries. As Roche 454 Sequencing Platform has a high base error rate, organelle genomes were also sequenced using the Roche 454 FLX + and Illumina MiSeq platforms (Personal Biotechnology, Shanghai, China). Genome sequences were assembled by Newbler Assembler Software Version 2.8 (454 Life Sciences, Branford, USA). Contig gaps were filled by sequences obtained from Sanger sequencing of PCR products amplified with primers designed based on contig end sequences.

BLASTX, BLASTN (http://blast.ncbi.nlm.nih.gov/Blast.cgi), and tRNA-SE [62] were used to identify mitochondrial and chloroplast rRNA, tRNA and genes. ORF Finder (http://www.ncbi.nlm.nih.gov/gorf/gorf.html) was used to screen hypothetical proteins longer than 100 amino acids by default parameter. Blast2seq (https://blast.ncbi.nlm.nih.gov/Blast.cgi) was used to perform sequence alignment to find the syntenic region of mitochondrial genomes. The presence of transmembrane domain in each hypothetical protein was performed with online TMHMM server v.2.0 (http://www.cbs.dtu.dk/services/TMHMM/). Phylogenetic analysis of candidate CMS-associated genes in Brassicaceae was performed by MEGA 7.0 [63]. DOGMA was used to visualize data and information of the mitochondrial genome of *Nsa* CMS line and its maintainer line [64]. Progressive Mauve was used for multiple alignments among the mitochondrial genomes in Brassica [65]. The Genbank accession numbers of protein sequences involved in this study are listed in Additional file 3.

## Supplementary information

**Additional file 1. **Comparative analysis of *Nsa* CMS, Zhongshuang 4, and *S. arvensis* chloroplast (cp) genomes. (A) Comparison between *Nsa* CMS cp genome (vertical axis) and Zhongshuang 4 cp genome (horizontal axis) indicated that the nucleotide sequences of the syntenic region are well conserved. (B) Alignment of *Nsa* CMS cp genome (vertical axis) and *S. arvensis* cp genome (horizontal axis). Apart from SNPs, they were consistent and no rearrangement was found.

**Additional file 2.** Primers for verification of mitochondrial genome recombination.

**Additional file 3.** Sequence information of genes involved in phylogenetic tree construction.

## Data Availability

The datasets generated during the current study are available in the Genbank with accession numbers MN443182 and MN428072 (mitochondrial) and MN428073 and MN428074 (chloroplast) genome sequences, respectively. The Genbank accession number of proteins and genomes used in the study are listed in the Additional file 2 and Additional file 3.

## Abbreviations

CMS: Cytoplasmic male sterility
IR: Inverted repeat
LSC: Long single copy
ML: Maximum likelihood
NGS: Next-generation sequencing
ORFs: Open reading frames
SNPs: Single nucleotide polymorphism site
SSC: Short single copy
