# Supplementary material for: Organelle genome composition and candidate gene identification for Nsa cytoplasmic male sterility in Brassica napus
Source: BMC Genomics. 2019 Nov 6;20:813. doi: 10.1186/s12864-019-6187-y (PMC6836354; doi:10.1186/s12864-019-6187-y)
Supplement: Supplementary file 1 — Additional file 1. Comparative analysis of Nsa CMS, Zhongshuang 4, and S. arvensis chloroplast (cp) genomes. (A) Comparison between Nsa CMS cp genome (vertical axis) and Zhongshuang 4 cp genome (horizontal axis) indicated that the nucleotide sequences of the syntenic region are well conserved. (B) Alignment of Nsa CMS cp genome (vertical axis) and S. arvensis cp genome (horizontal axis). Apart from SNPs, they were consistent and no rearrangement was found. [file 12864_2019_6187_MOESM1_ESM.docx]

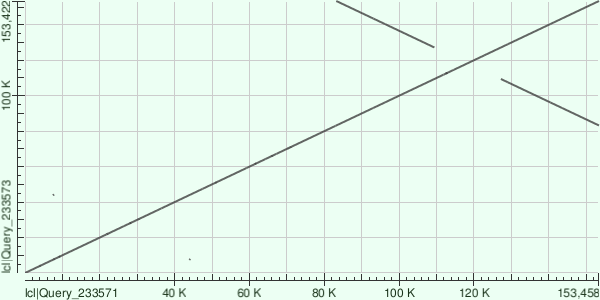

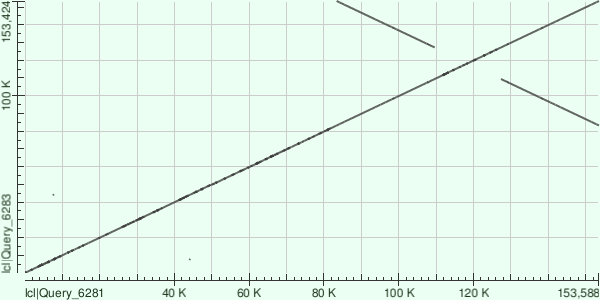


A

B

*Nsa* CMS cp

*Sinapis arvensis* cp

**Figure S1** Comparative analysis of *Nsa* CMS, Zhongshuang 4, and *S. arvensis* chloroplast (cp) genome. (A) Comparisons between *Nsa* CMS cp genome (vertical axis) and Zhongshuang 4 cp genome (horizontal axis) indicated that the nucleotide sequences of the syntenic region are well conserved. (B) Alignment of *Nsa* CMS line cp genome (vertical axis) and *S. arvensis* cp genome (horizontal axis). Apart from SNPs, they were consistent and no rearrangement was found.
